# Optimization of In Vivo Electroporation Conditions and Delivery of DNA Vaccine Encoding SARS-CoV-2 RBD Using the Determined Protocol

**DOI:** 10.3390/pharmaceutics14112259

**Published:** 2022-10-22

**Authors:** Denis Nikolaevich Kisakov, Lyubov Alexandrovna Kisakova, Maria Borisovna Borgoyakova, Ekaterina Vladimirovna Starostina, Oleg Svyatoslavovich Taranov, Elena Konstantinovna Ivleva, Oleg Viktorovich Pyankov, Anna Vladimirovna Zaykovskaya, Dmitry Nikolaevich Shcherbakov, Andrey Pavlovich Rudometov, Nadezda Borisovna Rudometova, Natalia Vyacheslavovna Volkova, Vadim Nikolaevich Gureev, Alexander Alexeyevich Ilyichev, Larisa Ivanovna Karpenko

**Affiliations:** State Research Center of Virology and Biotechnology VECTOR, Rospotrebnadzor, 630559 Koltsovo, Russia

**Keywords:** DNA vaccines, in vivo, electroporation, protocol optimization, RBD, SARS-CoV-2 receptor-binding domain, immune response

## Abstract

Vaccination against SARS-CoV-2 and other viral infections requires safe, effective, and inexpensive vaccines that can be rapidly developed. DNA vaccines are candidates that meet these criteria, but one of their drawbacks is their relatively weak immunogenicity. Electroporation (EP) is an effective way to enhance the immunogenicity of DNA vaccines, but because of the different configurations of the devices that are used for EP, it is necessary to carefully select the conditions of the procedure, including characteristics such as voltage, current strength, number of pulses, etc. In this study, we determined the optimal parameters for delivery DNA vaccine by electroporation using the BEX CO device. BALB/c mice were used as a model. Plasmid DNA phMGFP was intramuscular (I/M) injected into the quadriceps muscle of the left hind leg of animals using insulin syringes, followed by EP. As a result of the experiments, the following EP parameters were determined: direct and reverse polarity rectangular DC current in three pulses, 12 V voltage for 30 ms and 950 ms intervals, with a current limit of 45 mA. The selected protocol induced a low level of injury and provided a high level of GFP expression. The chosen protocol was used to evaluate the immunogenicity of the DNA vaccine encoding the receptor-binding domain (RBD) of the SARS-CoV-2 protein (pVAXrbd) injected by EP. It was shown that the delivery of pVAXrbd via EP significantly enhanced both specific humoral and cellular immune responses compared to the intramuscular injection of the DNA vaccine.

## 1. Introduction

Nucleic acid vaccines are a promising alternative platform for vaccine development, including those against COVID-19 [1]. The spread of SARS-CoV-2 has stimulated research into the development of new vaccines, including those based on mRNA and DNA [2,3]. mRNA vaccines were among the first to be licensed for use against this virus [2,3]. In 2021, a DNA vaccine developed by the Indian company Zydus Lifesciences Limited, called ZyCoV-D, was also approved for this purpose [4,5].

DNA vaccines have several advantages: similarly to recombinant viral vectors and mRNA vaccines, they induce T-cell-mediated and humoral immunity while having a relatively good safety profile. A nucleic acid vaccine is a platform that does not induce anti-vector immunity, making it suitable for vaccine regimens that include both priming as well as boosters [6,7,8]. The manufacturing of plasmid DNA is considerably faster and easier than the manufacturing of live-attenuated or subunit vaccines. Manufacturing of plasmid DNA can be conducted safely, especially compared to killed pathogenic vaccines. DNA vaccines can be quickly adapted to new targets, and they are stable at room temperature, which is an advantage over mRNA vaccines that require storage at low temperatures [1,6].

A DNA vaccine can be designed to increase the breadth of immune responses and potentially increase pathogen coverage. Synthetic consensus immunogens and polyepitope and mosaic immunogens are being developed on the DNA platform to create “universal” vaccines for simultaneous action against several different but related strains of these pathogens [9,10,11,12].

The main disadvantage of DNA vaccines is their low immunogenicity when administered as naked DNA [6,7,8]. A wide range of strategies were developed in attempts to increase the immunogenicity of DNA vaccines, including packaging in liposomes and in polycationic polymers, gene gun delivery, needleless injectors, and EP [4,13,14,15]. Electroporation is a very attractive delivery method that can increase the immunogenicity of DNA vaccines by two or three orders of magnitude [16,17].

The question of the choice of delivery system is one of the key issues on which the efficacy of a DNA vaccine depends. Zydus Cadila uses the injection delivery method for ZyCoV-D. For the delivery of several DNA vaccines against COVID-19 in clinical trials, the EP method was used [18,19,20].

Inovio Pharmaceuticals demonstrated that the INO-4800 DNA vaccine against SARS-CoV-2 induced a balanced immune response in volunteers characterized by both functional antibodies and T-cell responses in phase 1 clinical trials [19,20,21].

The EP procedure involves the generation of direct-current electrical pulses capable of causing the reversible destabilization of the cell lipid membrane, which leads to the effective delivery of nucleic acids (injected intramuscularly) from the intercellular space to the intracellular environment [22]. Using EP, it is possible to increase the delivery of plasmids and gene expression by 100–1000 times compared to the delivery of DNA without EP [12].

The EP procedure involves the generation of electrical pulses, or a direct current, capable of causing the reversible destabilization of the lipid membranes of cells, which leads to the efficient delivery of nucleic acids from the intercellular space to the cells [22]. Over the past two decades, the technique has evolved from an experimental technique to one that is now being used in several clinical trials to deliver nucleic acids, including DNA vaccines against COVID-19 [23,24,25].

However, according to the literature, although EP delivery may generally improve DNA vaccines’ immunogenicity compared to other methods, there are significant differences in the immune responses elicited depending on the pulse patterns, voltage–current and field strengths, electrode configurations, and impedance of target tissues. Electrode shape, size, and DNA vaccine formulation (optimized sequence, dose, concentration, and buffers) also play a critical role in the induction of immune responses and may need to be optimized depending on the particular vaccine target specifications for immunogenicity and efficacy [12].

A few companies manufacture electroporation devices for in vivo nucleic acid electrotransfer, such as Inovio Pharmaceuticals, BTX, BEX CO, etc. These devices are used in many scientific studies, including clinical trials [18,19,20,21,26,27,28]. However, data presented in the literature indicate some variability in immune responses for different DNA constructs and different EP devices [12]. Not all EP device and DNA vaccine combinations are likely to lead to the same outcome, due to their different design and delivery parameters.

In this study, we determined the optimal parameters for DNA vaccine delivery using the BEX CO device. We used phMGFP, encoding a green fluorescent protein, as a model plasmid. Two criteria were used in the search for the optimal protocol: first, minimization of damage to mouse muscle cells as a result of EP; second, the effective expression of GFP at the injection site.

The chosen protocol was used to immunize mice with a DNA vaccine encoding the receptor-binding domain (RBD) of the SARS-CoV-2 spike protein (pVAXrbd).

## 2. Materials and Methods

### 2.1. Plasmids

To develop the EP protocol, we used the phMGFP plasmid encoding the green fluorescent protein GFP (E6421, Promega, Madison, Wisconsin, USA) as a model.

The design of the DNA vaccine pVAXrbd was described earlier [29]. Briefly, to amplify the fragment encoding RBD protein S (320V–542 N) and signal sequence 176 (MMRTLILAVLLVYFCATVHC) at the 5′-end, primers were used: 5’-TAATACGACTCACTATAGGCTAGCCT-3′ (forward) and 5′-AAAAAAAGCGGCCGCTCATTAGTTGAAGTTCACGCATTTGTTCTTC-3′ (reverse),—and a matrix containing the indicated fragment (kindly provided by the Laboratory of Immunochemistry of the FBSI SRC VB “Vector” of Rospotrebnadzor). The amplification product was inserted into the pVAX1 vector (Thermo Fisher Scientific, Waltham, MA, USA) at the NheI and NotI sites. The structure of the resulting construct was confirmed by sequencing using the Sanger method at the Genomics Center for Collective Use (Novosibirsk, Russia). The resulting construct was designated pVAXrbd.

Plasmids phMGFP and pVAXrbd were amplified in *E. coli* Stbl3 and purified using the EndoFree Plasmid Giga kit (Qiagen, Hilden, Germany). Endotoxin level was determined using the LAL test according to the manufacturer’s protocol (Charles River, United States). Endotoxin content in the final preparation was not higher than 4 units (EU) per a single dose (100 µg DNA), which is lower than the threshold level accepted for vaccine preparations.

### 2.2. Animals

Eight-week-old BALB/c mice from the Nursery of the SRC VB «VECTOR» (State Research Center of Virology and Biotechnology VECTOR, Rospotrebnadzor, Koltsovo, Novosibirsk region, Russia) were housed under a 12  h/12  h light–dark cycle with ad libitum access to water and food. Experiments were carried out in compliance with the bioethical principles adopted by the European Convention for the Protection of Vertebrate Animals Used for Experimental and Other Scientific Purposes (Strasbourg, 1986) and the Order of the Ministry of Health of the Russian Federation of 23 August 2010, “Establishment of the Rules of Laboratory Practice” No. 708n. Animal manipulations were approved by the Bioethics Committee of the SRC VB VECTOR (Permit Number: SRC VB “Vector”/10-09.2020).

### 2.3. Selection of Optimal Parameters for EP

In a universal protocol EP selection experiment, we used the phMGFP plasmid encoding the green fluorescent protein GFP (Promega, Madison, WI, USA) as a model. To select the optimal protocol for the delivery of the phMGFP plasmid, there were 6 groups of mice, consisting of 5 animals. The plasmid was injected in the left hind leg muscle of mice (30 μg in 40 μL of PBS/dose/mouse) using insulin syringes with 29G needles. After injections, EP was performed using a CUY21 EDIT II in vivo electroporator (BEX Ltd, Japan) and an LF 650P5 5 mm tweezer electrode (BEX Co., Ltd., Tokyo, Japan).

Two approaches to the formation of a direct current pulse were used: exponential and rectangular. The voltage and current varied in the range from 100 to 12 V and from 300 to 45 mA, the pulses were transferred from 3 to 5. Before each EP, the complex resistance of the electrodes and vessels was checked.

Possible combinations of pulse parameters were applied to the electrodes: 3 voltage pulses of 30 ms or 40 ms at a voltage of 6 V or 50 V. The pulses were applied at intervals of 900, 950, or 1000 ms with opposite (±) polarity.

Details of all EP modes are shown in Table 1.

After in vivo EP, the second day, histopathology changes were assessed by analyzing a sample of muscle tissue of the mouse left hind paw.

GFP fluorescence was assessed on the third day after EP using UV microscopy of thin samples of animal muscle tissue using an Olympus fluorescent microscope (Olympus Life Science, Tokyo, Japan) with an exposure period of 550 ms.

To quantify GFP fluorescence, we performed computer processing to determine the intensity of green fluorescent protein fluorescence in muscle sections of BALB/c mice using the conventional value of GFP + fibers obtained as the number of pixels of green fluorescent protein spectrum in relation to the entire image. To calculate this value, we obtained a histogram of the image and compiled a table of relative values from the distribution of pixel color intensity in the image.

### 2.4. Histological Studies

For light-optical study, the muscle tissue sections of the mouse left hind leg were fixed in 10% buffered formalin solution (Biovitrum, St. Petersburg, Russia) for 48–72 h, washed with running tap water for 10 min. The same processing of the material was carried out according to the standard method: successive dehydration in alcohol with increasing concentration, impregnation with a xylene–paraffin mixture in a Tissue Tek VIP 6 AI vacuum histoprocessor (Sakura, Tokyo, Japan), and pouring into paraffin blocks. Paraffin sections 4–5 µm thick were prepared using an automatic rotary microtome HM-360 (Thermo Fisher Scientific Microm International GmbH, Breman, Germany), Accu-Cut SRM (Sakura Finetek, Japan). Sections were stained with hematoxylin and eosin and then covered with a protective film in Tissue Tek Film automatic stain (Sakura Finetek, Tokyo, Japan). Microphotography was carried out using an Imager Z1 microscope (Zeiss, Oberkochen, Germany).

### 2.5. DNA Immunization of Mice

To determine the most optimal protocol, each mouse received a single injection of phMGFP (30 μg/40 μL PBS dose) into the muscle of the left hind leg (quadriceps muscle). Mice were first placed in an induction chamber and then moved under anesthesia masks. Hair was removed from the hairy part of the paw using depilatory gel. Before intramuscular injection (I/M) of plasmids into the quadriceps muscle, the skin was treated with 70% ethanol. Plasmid was injected using insulin syringes with 29G needles, followed by EP using a CUY21 EDIT II in vivo electroporator (BEX Co., Ltd., Tokyo, Japan) and a LF 650P5 5 mm forceps electrode (BEX Co., Ltd., Tokyo, Japan).

To evaluate the immunogenicity of the plasmid pVAXrbd, 3 groups of mice consisting of 6 animals were immunized as follows: the first group (pVAXrbd) was injected with pVAXrbd intramuscularly at a dose of 100 µg/100 µL, the second group (pVAXrbd+EP) was injected intramuscularly with 100 µg/100 µL followed by EP with parameters from protocol No. 4 (Table 1). The third group consisted of non-immunized animals.

For primary immunization, the first injection was performed according to the standard immunization protocol, and the procedure was repeated on day 21. On day 10 after the second injection, mice were bled, killed, and their blood and spleens were collected for further analysis of plasmid pVAXrbd immunogenicity. The sera were separated from the cellular elements by centrifugation (10,000 rpm, 10 min), warmed to inactivate the complement system (30 min, 56 °C).

### 2.6. ELISA Analysis

As an antigen for the enzyme-linked immunosorbent assay (ELISA), the RBD protein produced in CHO-K1 cells and purified using affinity and ion-exchange chromatography methods (protein purity > 98%) was used [30]. RBD protein (1 µg/mL in 2 m urea) was adsorbed onto 96-well plates (Greiner Bio-One, Kremsmünster, Austria) at 4 °C overnight, then the plate was washed in PBS with 0.05% Tween 20 (PBST) and blocked with 1% casein solution in the same buffer for 60 min at room temperature. Then, sera were added to the wells in threefold serial dilutions, starting from 1:50, in blocking solution, and incubated for 60 min at room temperature. The plate was washed, and horseradish-peroxidase-conjugated rabbit anti-mouse IgG antibodies (Sigma, St. Louis, MO, USA) were added and incubated for 60 min at room temperature. The substrate 3,3’,5,5’-tetramethylbenzidine (TMB) (Amresco, Dallas, TX, USA) was introduced into the wells of the plate washed with PBST. Optical density was measured at a wavelength of 450 nm using a ChroMate-4300 microplate reader (Awareness Technology Inc., Palm City, FL, USA). Serum dilution was taken as the titer in ELISA, at which the optical density value was more than two times higher than that for the negative control (blocking buffer was added to the wells instead of serum).

### 2.7. Virus Neutralization

Vero E6 cells were used in the experiment (cells of the epithelium of the kidney of the African green monkey) (collection of SRC VB “Vector” Rospotrebnadzor, RF). The cells were cultured in a DMEM medium (Gibco, Thermo Fisher Scientific, Waltham, MA, USA) with L-glutamine, with 10% fetal calf serum (Gibco, Thermo Fisher Scientific, USA) and antibiotic-antimycotic (Gibco, Thermo Fisher Scientific, USA) at 37 °C, 5% CO_2_.

Coronavirus 2019-nCoV strain nCoV/Victoria/1/2020 was used (State collection of pathogens of viral infections and rickettsiosis SRC VB "Vector" of Rospotrebnadzor, RF). Studies using the SARS-CoV-2 virus were performed in laboratories with BSL-3 containment. The infectious titer of the virus was determined by titration on a monolayer of cell culture. Successive ten-fold dilutions of the virus were introduced into the wells with the cell culture. After 4 days, the result was considered for the presence of cytopathic effect (CPE) after staining with gentian violet (Sigma, USA) solution. For cell staining, 150 μL of 0.2% gentian violet solution was added to each well of the plate (1 g of gentian violet was dissolved in 20 mL of 96% ethanol, 120 mL of 40% formalin, and 350 mL of Hanks’ solution). After 30 min, the liquid from the wells was removed and washed with water. The specific defeat of the cell culture monolayer was considered as CPE. The 50% tissue culture infectious dose (TCID50) was calculated using the Reed–Mench formula and expressed in lg TCID50/mL [31].

Serial two-fold dilutions of serum samples were prepared, starting with 1:10 to 1:1280. Serums were diluted in DMEM medium with glutamine and antibiotics. A mixture of serum dilutions with virus in dose 2 × 100 TCID50 in equal volumes was prepared. The mixture was incubated for 1 h at room temperature, then added to the 96-well plate with a monolayer of Vero E6 cell culture [32]. The plates were incubated for four days at 37 °C, 5% CO_2_, stained with gentian violet solution. The neutralization titers were defined as the reciprocal of the highest dilution at which a 50% reduction was observed relative to the uninfected control wells.

### 2.8. Isolation of Splenocytes

The spleens were successively minced on nylon cell filters with a pore diameter of 70 and 40 µm (BD Falcon^TM^, Tucson, AZ, USA). After erythrocyte lysis with lysis buffer (Sigma, USA), splenocytes were washed twice in complete RPMI medium and placed in 1 mL of RPMI medium with 2 mm L -glutamine, gentamicin (50 μg/mL) and 10% fetal bovine serum (FBS) (Thermo Fisher Scientific, USA). Cells were counted using a TC 20™ automatic cell counter (Bio-Rad, Hercules, CA, USA).

### 2.9. IFN-γ ELISpot Analysis

The analysis was carried out using kits (the name of the kit should be written) Mabtech (United States, Cincinnati) in accordance with the manufacturer’s recommendations. Splenocytes isolated from immunized animals were stimulated using a pool of 9 SARS-CoV-2 RBD peptides (20 µg/mL of each peptide) recognized by major histocompatibility complex (MHC) class I (H-2-Dd, H-2-Kd, H-2-Ld) and class II molecules (H2-IAd, H2-IEd) for BALB/c mice. Peptides were synthesized by AtaGenix Laboratories (Wuhan, China), peptide purity > 80%. The number of IFN-γ-producing cells was counted using an ELISpot analyzer from Carl Zeiss (Oberkochen, Germany).

### 2.10. Statistics

Data were analyzed using GraphPad Prism 9.0 software (GraphPad Software, Inc., San Diego, CA, USA). The results are expressed as a median with a range. The data were analyzed using non-parametric tests. Between-group differences in immune responses were assessed using non-parametric, one-way Kruskal–Wallis analysis of variance, adjusted for multiple comparisons and Dunn’s statistical hypothesis test. *p* < 0.05 was considered statistically significant. Comparisons were not statistically significant unless otherwise noted.

## 3. Results

### 3.1. Optimization of In Vivo EP Conditions

The selection of optimal parameters was performed according to two criteria: first, the traumaticity of the procedure should be minimal; second, stable biosynthesis of the green fluorescent protein from the phMGFP matrix should be observed.

Two different approaches to the formation of direct-current (DC) pulses can be applied: exponential and rectangular. The first type of pulse is characterized by a short burst of electric voltage followed by an exponential decay, while the second has a sharper, truncated decay, which causes it to take the form of a rectangle. Based on our experiments, the exponential pulse is ineffective and traumatic, so we decided to use rectangular pulses of different voltages and currents. Variants of protocols with different efficiencies are presented in Table 1.

To assess the extent of damage, a histological analysis of skin preparations with adjacent muscles was performed; samples were taken at the injection site on day 2 after EP without waiting for complete tissue recovery after the procedure.

**Figure 1:**
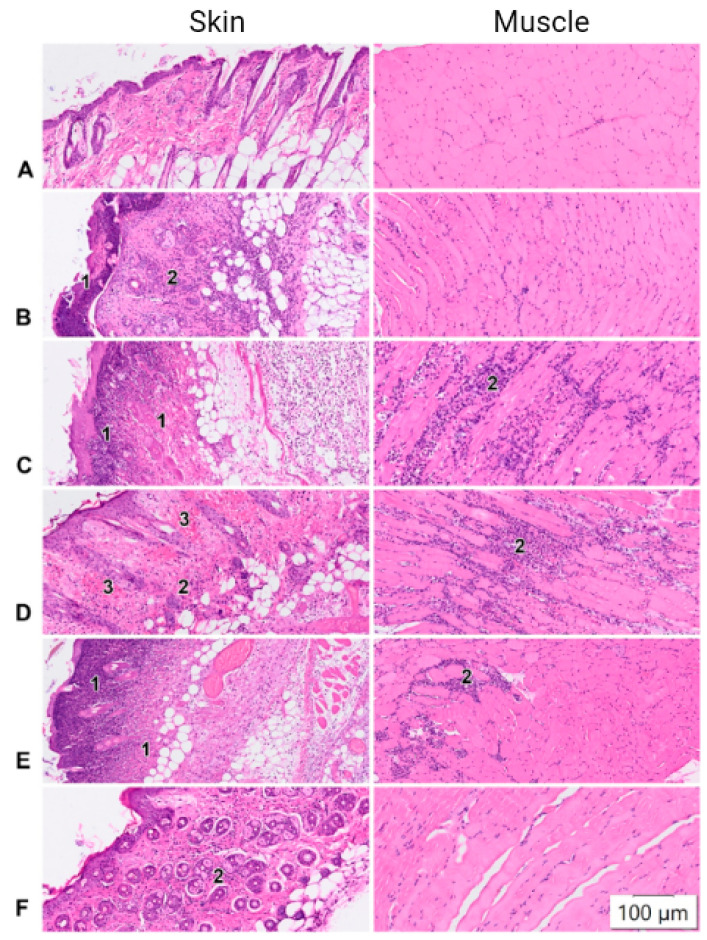
Light micrographs of murine tissue samples showing histological changes with different EP protocols (see Table 1). Sections stained with hematoxylin and eosin: (**A**) Control mice, (**B**) Protocol No. 1; (**C**) Protocol No. 2; (**D**) Protocol No. 3; (**E**) Protocol No. 4; (**F**) Protocol No. 5. Mice injected with the GFP plasmid were electroporated and tissue was cut into 1 mm thick sections. Designations: 1—necrosis, 2—inflammatory infiltration, 3—plethoric vessel microcirculation, erythrocyte diapedesis.

The degree of histopathological change depended on the protocol used (Figure 1B–E). In the group of animals immunized according to Protocol No. 1, pathohistological changes were reduced to moderate mixed inflammatory infiltration throughout the depth of the dermis and hypodermis and necrotic changes at the epidermis level. A single acanthosis occurred in one animal (Figure 1B). In the group of animals vaccinated according to Protocol No. 2, after EP, we observed pronounced acute inflammatory (neutrophilic) infiltration, with the foci of necrosis spreading to the entire depth of the skin flap (derma, hypoderma), with transition to the underlying muscle tissue and myocytolysis in some animals and necrosis of skin appendages (hair follicles, sebaceous glands). This group was also characterized by pronounced edema at the level of the reticular dermis and hypodermis (Figure 1C). When using Protocol No. 3, the mice showed focal transdermal necrosis in the injection area and moderate, diffuse neutrophil–lymphocytic/neutrophil–histiocytic infiltration throughout the dermis and hypodermis, sometimes with a transition to the muscle tissue. The blood vessels of the papillary layer of the dermis were full (Figure 1D). In mice immunized using Protocol No. 4, moderate inflammatory infiltration, mainly by neutrophils, was recorded at the level of the subcutaneous fat layer, along with muscle tissue and focal transdermal necrosis in some cases (Figure 1E). The use of Protocol No. 5 caused mild focal inflammatory infiltration in the dermis, however, we did not observe GFP fluorescence in the muscle using this protocol, indicating inefficient delivery of the phMGFP plasmid (Figure 1F).

**Figure 2:**
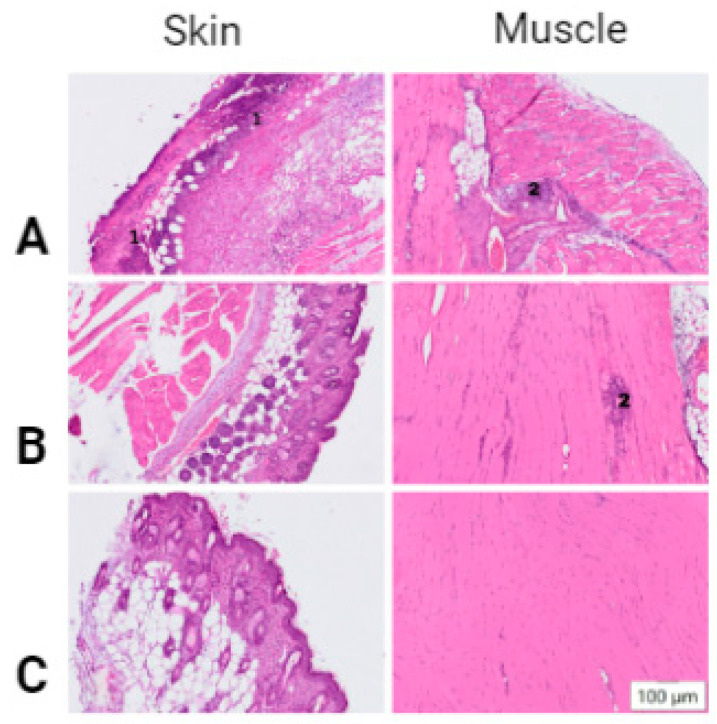
Light micrographs of murine tissue samples showing histological changes in control mice immunized via EP with PBS, I/M injection phMGFP (without EP) and mouse tissue sections stained with hematoxylin and eosin: (**A**) (Protocol No. 4), (**B**) injection phMGFP (without EP), (**C**) Control. Designations: 1—necrosis, 2—inflammatory infiltration.

In order to determine whether the histopathological changes were the result of the electroporation procedure or I/M injection of DNA (without EP), we performed the following control experiments. EP was performed using the conditions of Protocol No. 4 after I/M injection of PBS, and I/M injection of phMGFP DNA was also performed (without EP). Histological analysis of tissue at the injection site showed that the EP procedure, when administered with PBS, caused mild pathological changes in the skin and a small focus of inflammatory cell infiltration in the muscle (Figure 2B). The histological picture was consistent with the introduction of PBS+phMGFP using EP. With I/M administration of phMGFP without EP, no pathological changes in the skin were registered, and a small focus of neutrophilic infiltration was observed in the muscles at the injection site.

**Figure 3:**
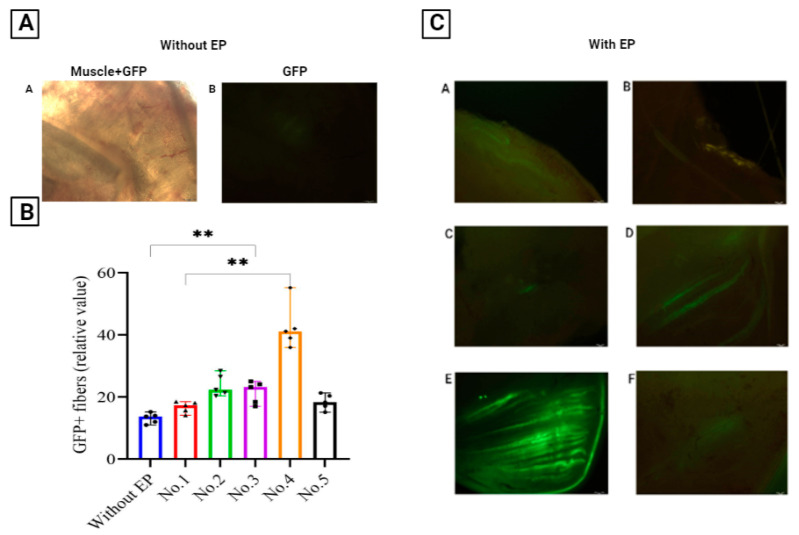
Fluorescence micrographs of muscle tissue imaged 3 days after immunization with GFP reporter plasmid. The injected muscle was harvested and then sectioned into 1 mm thick sections to visualize GFP expression: (**A**)—phMGFP was delivered by intramuscular injection without EP: (A)—visualization of GFP expression using either a white light lamp (muscle + GFP) or (**B**)—a UV light lamp (GFP). (**B**)—Graphical representation of fluorescence intensity using computer signal processing. Significance was assessed with the nonparametric one-way Kruskal–Wallis analysis of variance adjusted for multiple comparisons and Dunn’s statistical hypothesis testing (** *p* < 0.01). (**C**)—Representative images are shown after EP using different electroporation protocols: (**A**) Control mice (without EP); (**B**) Protocol No. 1; (**C**) Protocol No. 2; (**D**) Protocol No. 3; (**E**) Protocol No. 4; (**F**) Protocol No. 5.

GFP fluorescence was assessed on day 3 after EP using UV microscopy of thin slices of animal muscle tissue, since this is the period when intensive GFP fluorescence begins. Representative micrographs are shown in Figure 3C(A–F).

As shown in Figure 3B, the greatest number of fibers with GFP fluorescence was registered in mice immunized using Protocol No. 4. The GFP signal intensity in this group of mice was statistically significantly different from that in the other groups of animals.

It should be noted that moderate histopathological changes were also observed in group 4 (Figure 1E). Based on this, we can argue that Protocol No. 4 is the most preferable in comparison with the other protocols that we studied. The parameters of Protocol No. 4 were direct and reverse polarity rectangular impulse DC in count three pulses, 12 V, 30 ms, and 950 ms intervals, with a current limit of 45 mA.

**Figure 4:**
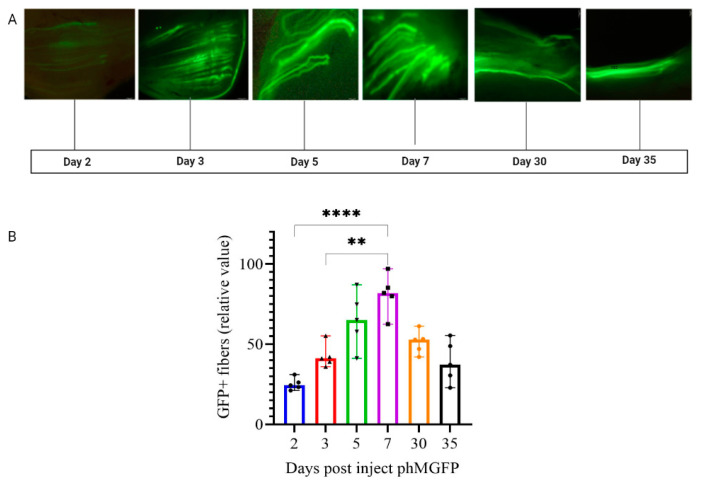
(**A**) Fluorescence micrographs of mouse tissue imaged 2, 3, 5, 7, 30, and 35 days after delivery of phMGFP by EP. The injected muscle was harvested and then sectioned into 1 mm thick sections to visualize GFP expression. (**B**) Graphical representation of fluorescence intensity using computer signal processing. Significance was assessed with the non-parametric one-way Kruskal–Wallis analysis of variance adjusted for multiple comparisons and Dunn’s statistical hypothesis testing (** *p* < 0.01, **** *p* < 0. 0001).

The formation of an adaptive immune response requires long-term and stable immunogen expression, so we decided to examine the duration of GFP expression in the muscles of BALB/c mice at control points 2, 3, 5, 7, 30, and 35 days after EP using Protocol No. 4, as shown in Figure 4.

As shown in Figure 4, GFP expression was detected on day 2 and peaked on day 7 after EP of the phMGFP plasmid using Protocol No. 4.

### 3.2. Humoral Immune Response in Mice

To assess humoral immunity in animals immunized twice with pVAXrbd administered either I/M or by EP on days 0 and 21, at the end point (day 31, 10 days after the second immunization), serum samples were taken and tested by enzyme-linked immunosorbent assay (ELISA) for the presence of antibodies specifically recognizing the RBD protein, as well as the ability of sera to neutralize live virus.

**Figure 5:**
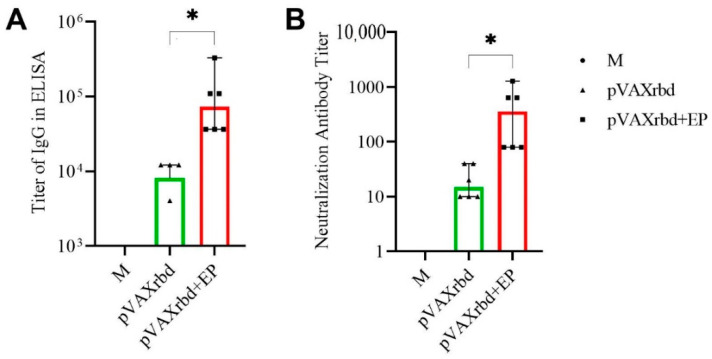
Humoral response in BALB/c. M—control (non-immunized) mice; pVAXrbd—mice immunized with naked plasmid, pVAXrbd+EP—mice immunized with plasmid using EP. (**A**) The titers of RBD-specific IgG to SARS-CoV-2 RBD were determined by ELISA. (**B**) The virus-neutralizing activity of sera from mice immunized with pVAXrbd+EP and pVAXrbd was determined using the SARS-CoV-2 nCoV/Victoria/1/2020 strain (100 TCID50). In panels (**A**,**B**), data are presented as median with a range of reciprocal titers. Significance was assessed with the nonparametric one-way Kruskal–Wallis analysis of variance adjusted for multiple comparisons and Dunn’s statistical hypothesis testing (* *p* < 0. 05).

The results of the ELISA of sera are shown in Figure 5A.

According to the results of the RBD-specific ELISA, a statistically significant (*p* <0.05) 16-fold increase in the mean titer of specific antibodies in mice immunized with pVAXrbd using EP was noted, compared with naked pVAXrbd.

The data demonstrate that the administration of the pVAXrbd DNA vaccine by EP induced a significantly higher level of antibody synthesis compared to the intramuscular naked vaccine.

An important indicator of a vaccine’s effectiveness is its ability to induce antibodies that can neutralize the live virus. In our study, sera from mice immunized with the pVAXrbd DNA vaccine demonstrated the ability to neutralize the nCoV/Victoria/1/2020 strain of the SARS-CoV-2 virus on cell culture in vitro in a virus neutralization reaction (Figure 5B). At the same time, the pVAXrbd+ EP group had a titer of virus-neutralizing antibodies more than 20 times higher than the I/M injection of pVAXrbd (*p* < 0.05).

The results of virus neutralization analysis correlated with the results of the ELISA: the sera of animals immunized with pVAXrbd using EP showed higher activity in inhibiting the cytopathic effect of the virus.

### 3.3. Analysis of the T Cell Immune Response 

Splenocytes were isolated from the spleens of immunized animals on day 31 after their first immunization with pVAXrbd to assess the cellular response using ELISpot. The response was assessed by the ability of splenocytes to secrete interferon-γ after specific stimulation, which was carried out using a pool of peptides from RBD protein.

**Figure 6:**
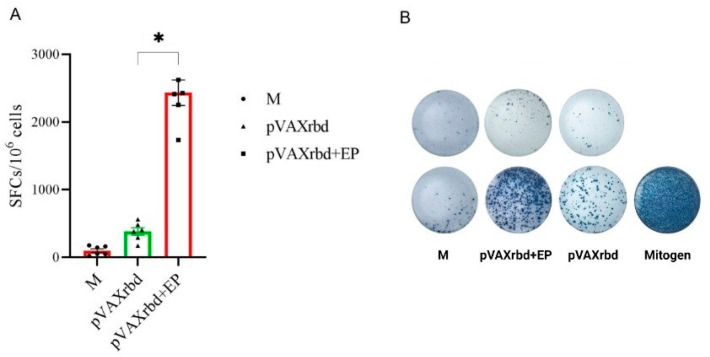
The results of the ELISpot assay of the SARS-CoV-2-specific T-cell-mediated response in immunized BALB/c mice. M—control (non-immunized) mice; pVAXrbd—mice immunized with naked plasmid, pVAXrbd+EP—mice immunized with plasmid using EP. (**A**) The number of cells expressing IFN-γ in response to stimulation with a pool of RBD-specific peptides, per 1 × 10^6^ splenocytes. (**B**) The representative images of ELISpot wells (top row: splenocytes not stimulated by peptides; bottom row: splenocytes stimulated with peptide pool or a mitogen). Significance was assessed with the non-parametric one-way Kruskal–Wallis analysis of variance adjusted for multiple comparisons and Dunn’s statistical hypothesis testing (* *p* < 0.05).

ELISpot analysis showed that the largest number of cells producing IFN-γ in response to stimulation with RBD-specific peptides was found in mice immunized using EP (Figure 6), indicating an increase in the post-vaccination T-cell response when using EP. These data correlate with an increase in the humoral immune response.

The average level of SFCs per 10^6^ splenocytes was 2432 for the pVAXrbd+EP group, and 381 for the pVAXrbd group immunized only with naked pVAXrbd. The higher level of IFN-γ-producing cells in the group immunized with the plasmid by EP was due to the fact that EP increased the efficiency of transfection due to the temporary destabilization of the lipid membrane, thereby increasing the uptake of the plasmid. In the group of mice immunized with pVAXrbd without EP, a lower response according to ELISpot data was recorded, which did not significantly differ from the cellular response found in the group of intact animals.

## 4. Discussion

According to the WHO (WHO: https://covid19.who.int/), as of 22 September 2022, there were a total of 610,866,075 confirmed cases of COVID-19 worldwide, including 6,510,139 deaths.

The most effective strategy to combat this epidemic is a large-scale vaccination program against COVID-19 [33].

The SARS-CoV-2 pandemic has stimulated the development of new vaccine platforms. Currently, mRNA and vector vaccines remain at the forefront, but DNA-based vaccines are also entering the market [5,34]. Several DNA vaccines are in preclinical and clinical trials [34], and the Indian company Zydus Cadila has developed ZyCoV-D, the world’s first DNA vaccine approved for human vaccination [4,5].

As already mentioned using EP, it is possible to increase the delivery of plasmids and gene expression by 100–1000 times compared with the delivery of DNA without EP [12]. However, although EP can generally improve the immunogenicity of DNA compared to other methods, there are significant differences in the immune responses elicited depending on the different device configurations, the nature of the pulse, the current strength and voltage, and the electrode configuration, shape, and size. The compositions of the DNA vaccine (sequence, dose, concentration, buffers) also play a crucial role in the induction of immune responses and may need to be optimized depending on the particular specifications of the vaccine with respect to immunogenicity and efficacy [12].

In the first stage of our study, we chose the optimal parameters for DNA vaccine delivery using the CUY21 EDIT II device and a LF 650P5 5 mm forceps electrode (BEX Co., Ltd., Japan). The plasmid phMGFP, encoding a green fluorescent protein, was used as a model plasmid. We used intramuscular injection and EP technology to deliver plasmid DNA.

According to many studies, skin and muscle are very attractive targets for plasmid DNA delivery. The skin is an immunological barrier that contains many immunocompetent antigen-presenting cells (APCs), such as Langerhans cells [17,35,36]. Intramuscular injection is preferable when a humoral response is required after DNA vaccine EP, or when both humoral and cellular levels of immune defense need to be activated [27]. Muscle tissue provides a mechanism for more effective plasmid DNA expression, thanks to myocytes organized into muscle fibers. For example, it was shown in a study by Vandermeulen, Galle et al. [37] that EP into muscle led to the highest expression of luciferase DNA. In addition, the syncytial nature of muscle fibers facilitates the spread of the transgene from one penetration site to many neighboring nuclei within the same fiber. Therefore, we chose intramuscular EP in our study.

Analyzing the literature, we found a published protocol for the delivery of the SHIV LentiDNA vaccine to mice and macaques using the CUY21 EDIT II electroporator (BEX Co., Ltd., Japan) [28]. The protocol included the following characteristics: preliminary shock pulses for 10 ms at 140 V and 5 pulses for 10 ms at 20 V; the current limit was 300 mA. The authors of the article noted that, despite the effectiveness of the vaccine, EP caused noticeable side effects [28]. We attempted to reproduce the conditions of this protocol for in vivo EP; however, when using the LF 650P5 electrode with a diameter of 5 mm, severe damage to the tissues of the skin and muscles of the mice occurred, followed by necrosis. Therefore, we decided to choose milder EP conditions.

Two criteria were used to determine the optimal protocol of EP: first, minimization of invasiveness of the procedure; second, the successful biosynthesis of the green fluorescent protein at the site of injection of phMGFP. The parameters and protocols used for the EP procedure are presented in Table 1.

Two criteria were used in the search for the optimal protocol: first, minimization of damage to mouse muscle cells as a result of EP; second, the effective expression of GFP at the injection site.

Two different approaches to the formation of DC pulses can be used for the EP method: exponential and rectangular. The first type of pulse is characterized by a short burst of electric voltage followed by an exponential decay, and the second by a sharper, truncated decay, so that it takes the form of a rectangle. A histological study of skin and muscle samples of mice after EP showed that the most pronounced pathohistological changes were observed in the animals subjected to EP with the use of Protocol No. 1 and No. 2 (Figure 1B, C). These tissue images showed large foci of necrosis, inflammatory infiltrates, and edema. At the same time, relatively weak GFP synthesis was observed in these samples, probably due to the significantly damaged muscle cells (Figure 2 and Figure 3C(B,C)). A moderate degree of skin and muscle tissue damage was observed in animals immunized according to Protocol No. 3 and No. 4 (Figure 1C,D). However, fluorescence in Protocol No. 3 was weaker than that in Protocol No. 4 (Figure 2 and Figure 3C(D,E)). Thus, the delivery of nucleic acids by an exponential pulse (Protocol No. 1) compared to a rectangular pulse (Protocol No. 3) was ineffective, because it caused severe post-traumatic traces in laboratory animals at a lower voltage. A normal histology picture was observed with Protocol No. 5, but no effective biosynthesis of the GFP protein was observed. The protocol meeting both criteria was Protocol No. 4, because it resulted in stable GFP protein fluorescence with moderate damage in laboratory animals (Figure 1E and Figure 3C(E)). 

The result of the histological analysis of tissues in the area of injection showed that the EP procedure with the introduction of PBS caused mild pathological changes in the skin and a small focus of inflammatory cell infiltration in the muscle (Figure 2B). The histological picture was consistent with the introduction of PBS + phMGFP DNA using EP. With I/M administration of phMGFP without EP, no pathological changes in the skin were registered, and a small focus of neutrophilic infiltration was observed in the muscles at the injection site. Analyzing these data, one can make the hypothesis that when plasmid DNA delivery is enhanced by electroporation, the area of neutrophil infiltration is increased due to the greater absorption of molecules and activation of protein synthesis.

An important factor in effective delivery, especially for vaccines based on nucleic acids, is the long-term synthesis of the target gene. In our study, we assessed expression at several checkpoints (days 2, 3, 5, 7, 30, and 35) after EP according to Protocol No. 4. Based on the data obtained, it was observed that the expression of the phMGFP plasmid reached a peak on day 7 and was maintained at a high level for a month. However, it then gradually began to decrease by day 35 (Figure 4A,B), which is a good indicator of the formation of adaptive immunity. The parameters of Protocol No. 4 were as follows: a rectangular direct and reverse-polarity DC current in three pulses, 12 V, 30 ms and 950 ms intervals, with a current limit of 45 mA.

After selecting the optimal conditions, we used the Protocol No. 4 to evaluate the immunogenicity of the DNA vaccine encoding the SARS-CoV-2 RBD protein (pVAXrbd). The design of pVAXrbd was described previously [29]. To enhance the synthesis of the RBD protein encoded by this plasmid, we optimized the codon composition of the target gene. To efficiently transport the protein from the cell, pVAXrbd includes a sequence encoding the initial leader sequence 176, a hybrid of the signal sequences of two actively secreted proteins: luciferase and fibroin [34]. Immunization of mice with pVAXrbd DNA was performed according to Protocol No. 4 using the CUY21 EDIT II device (BEX Co., Ltd., Japan).

Analysis of the humoral response using ELISA showed that the titer of RBD-specific antibodies in the group of mice immunized with pVAXrbd by EP was 1:109,350, which was 16 times higher than in the group of animals that received only the DNA vaccine I/M (titers 1:6750) (Figure 5A). When studying the cellular response, the highest number of cells (2433 spots/mln of splenocytes) producing IFN-γ in response to stimulation with peptides from the RBD protein was recorded in the group of animals immunized with pVAXrbd by EP. For comparison, in the control group with intramuscular injection of pVAXrbd, the number of cells was six times lower and amounted to 380 spots/mln of splenocytes (Figure 5).

Sera from the experimental groups demonstrated the ability to neutralize the SARS-CoV-2 nCoV/Victoria/1/2020 strain in cell culture in vitro (Figure 5B). In the pVAXrbd + EP group, the average neutralizing titer was 1:466, which was significantly higher than the titers shown in the pVAXrbd group (1:23). The data obtained show an increase in the efficiency of pVAXrbd DNA vaccine delivery using EP and the optimized Protocol No. 4.

Thus, the determined EP protocol allows for a significant enhancement in both the humoral and cell-specific immune responses of the pVAXrbd DNA vaccine encoding the RBD gene of SARS-CoV-2.

The level of immune response in mice immunized with pVAXrbd using the optimized protocol of EP was comparable to the responses to DNA vaccines against SARS-CoV-2 that are already approved for humans or are in clinical trials. Thus, when mice were immunized three times with the ZyCoV-D vaccine using a jet injector, the titer of IgG antibodies against the S protein in the blood serum of animals reached 1:28,000. The number of cells that responded to stimulation with peptides from the S in the ELISPOT assay was 200–300 SFC per 10^6^ splenocytes at immunizations of both 25 μg and 100 μg per dose [38]. 

Immunization of mice with the COVID-eVAX vaccine (at a dose of 20 μg–10 μg in each quadriceps muscle) by intramuscular injection and subsequent EP induced antibodies against the RBD protein in animals with an average titer of 1:70,000; the response in IFN-γ ELISPOT analysis was 360 SFC per 10^6^ splenocytes [14].

In the INO-4800 studies, single immunization of mice with the DNA vaccine (at doses of 2.5, 10, and 25 µg) was performed using the CELLECTRA device. The titer of anti-S antibodies in ELISA was 1:8000, and the titer of virus-neutralizing antibodies was 1:90 [39].

In our next studies, we plan to investigate the immunogenicity of the pVAXrbd DNA vaccine using EP and an optimized protocol in large laboratory animal models, including hamsters, guinea pigs, and nonhuman primates.

## 5. Conclusions

The number of positive results from studies of electroporation for the delivery of a DNA vaccine is steadily increasing, despite the possible difficulties. We expect our electroporation protocol to stimulate new research that achieves high efficiency through parameter selection. A major feature of our approach is the limitation of voltage and current, which allows us to provide an effective immune response without significant tissue damage at the injection site.

## Figures and Tables

**Table 1:** Different variants of EP protocols.

Protocol Number	1	2	3	4	5
Pulse mode	Exponential	Rectangular			
Type of impulse	±				
Voltage (V)	40	30	50	12	6
Current limit (mA)	300	200	150	45	45
Pulse time (ms)	30	40	30	30	30
Interval impulses (ms)	1000	900	900	950	900
Number of impulses	3	3	5	3	3
Injury	moderate infiltration	diffuse neutrophilic infiltration	neutrophilic-histiocytic infiltration	moderate infiltration	normal histological picture.
Level of GFP fluorescence	66	97	158	85	71

Note. Efficacy of phMGFP delivery was assessed on day 3 after EP by evaluating GFP fluorescence intensity in muscle samples using fluorescence microscopy.
